# Acaricidal Activity of Petroleum Ether Extract of Leaves of *Tetrastigma leucostaphylum* (Dennst.) Alston against *Rhipicephalus (Boophilus) annulatus*

**DOI:** 10.1155/2014/715481

**Published:** 2014-11-10

**Authors:** T. P. Adarsh Krishna, T. P. Ajeesh Krishna, N. D. Chithra, P. E. Deepa, U. Darsana, K. P. Sreelekha, Sanis Juliet, Suresh N. Nair, Reghu Ravindran, K. G. Ajith Kumar, Srikanta Ghosh

**Affiliations:** ^1^Department of Veterinary Pharmacology and Toxicology, College of Veterinary and Animal Sciences, Kerala Veterinary and Animal Sciences University, Pookode,Wayanad, Kerala 673 576, India; ^2^Department of Veterinary Parasitology, College of Veterinary and Animal Sciences, Kerala Veterinary and Animal Sciences University, Pookode,Wayanad, Kerala 673 576, India; ^3^Entomology Laboratory, Division of Veterinary Parasitology, Indian Veterinary Research Institute, Izatnagar, Uttar Pradesh 243 122, India

## Abstract

The acaricidal activity of the petroleum ether extract of leaves of *Tetrastigma leucostaphylum* (Dennst.) Alston (family: Vitaceae) against *Rhipicephalus (Boophilus) annulatus* was assessed using adult immersion test (AIT). The per cent of adult mortality, inhibition of fecundity, and blocking of hatching of eggs were studied at different concentrations. The extract at 10% concentration showed 88.96% inhibition of fecundity, 58.32% of adult tick mortality, and 50% inhibition of hatching. Peak mortality rate was observed after day 5 of treatment. Mortality of engorged female ticks, inhibition of fecundity, and hatching of eggs were concentration dependent. The LC_50_ value of the extract against *R. (B.) annulatus* was 10.46%. The HPTLC profiling of the petroleum ether extract revealed the presence of at least seven polyvalent components. In the petroleum ether extract, nicotine was identified as one of the components up to a concentration of 5.4%. However, nicotine did not reveal any acaricidal activity up to 20000 ppm (2%). Coconut oil, used as diluent for dissolving the extract, did not reveal any acaricidal effects. The results are indicative of the involvement of synergistic or additive action of the bioactive components in the tick mortality and inhibition of the oviposition.

## 1. Introduction 

Ticks are obligate haematophagous external parasites of domestic and wild fauna of animals. Heavy tick infestation on animals causes discomfort and annoyance that lead to reduction of milk production and weight gain. In addition, tick borne diseases continue to be a serious animal health problem, causing major economic loss to farmers. Chemical acaricides such as organophosphate compounds, synthetic pyrethroids, and amitraz are used for control of ticks [1–3]. The continuous application of these chemical acaricides results in acaricidal resistance, environmental pollution, residues in food, and toxicity to workers [4]. There is an urgent need of an alternative safe method for tick control. Herbal acaricides have many advantages over synthetic acaricides since they are eco-friendly and cheaper and are with minimum environmental and mammalian toxicity [5, 6]. Many plant extracts were analysed for their acaricidal effects in different laboratories of the world [7–10] including our laboratory [11–15]. The activities of plant extracts such as preventing blood feeding, molting, fecundity, and hatching of eggs [5] were reported in literature.

**Figure 1:**
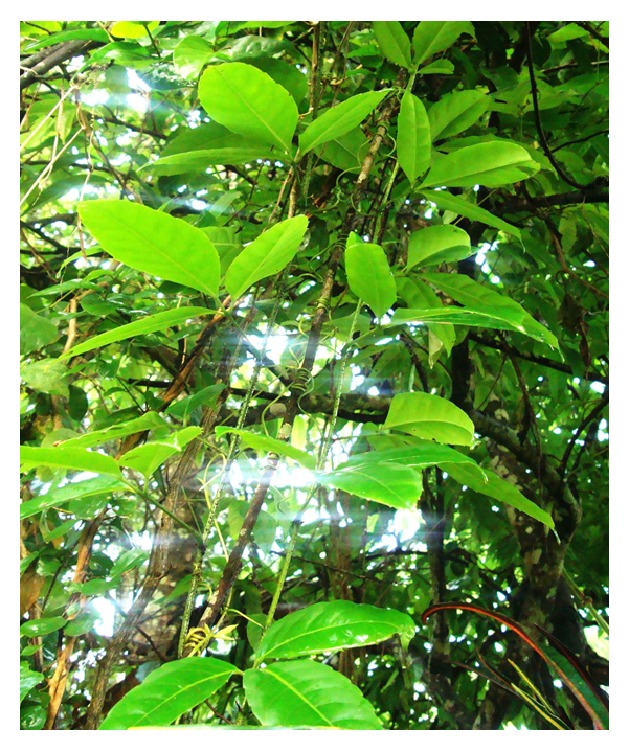
*Tetrastigma leucostaphylum* (Dennst.) Alston. (in wild) leaves are simple, defoliate, and trifoliate; all three types were found on the same plant; leaf 0–17 × 0–12; leaflets 4–13 × 2–6 cm; oblong-lanceolate; base cuneate; margin coarsely serrate, especially distally; apex acute; coriaceous; the laterals unequal sided; petiole to 8 cm long; warty-lenticellate; base swollen; petioules 5–14 mm long.

*Tetrastigma leucostaphylum *(Dennst.) Alston is a woody climber, which belongs to the family Vitaceae (Figure 1). It is an important ethnomedicinal plant used among the tribal folklore in Wayanad district. Currently, very few reports exist on the pharmacological properties of this plant. Therefore, the present investigation focuses on the acaricidal properties of petroleum ether extract of leaves of* T. leucostaphylum* against* R. (B.) annulatus*.

## 2. Materials and Methods 

### 2.1. Plant Material

*Tetrastigma leucostaphylum *(Dennst.) Alston (Vitaceae) leaves used in the present study were collected during May-June, 2012, from “Chendakuni” near Meenangadi, Wayanad, Kerala, India. A herbarium for morphological studies was prepared, identified, and authenticated by a botanist. A voucher specimen was deposited at the Department of Botany, Calicut University, Kerala (CALI- 6771).

### 2.2. Petroleum Ether Extract

The collected plant leaves were cleaned by washing in running water. The leaves were dried at room temperature for two weeks. The dried leaves (100 g) were powdered in a plant sample grinder at controlled temperature and extracted using petroleum ether in a Soxhlet extraction apparatus attached with a rotary vacuum evaporator (Buchi, Switzerland). Solvents were removed using rotary vacuum evaporator at 175 mbar at a temperature ranging from 40°C to 60°C.

### 2.3. High Performance Thin Layer Chromatography (HPTLC) Profiling

High performance thin layer chromatography (HPTLC) analysis was carried out on a HPTLC (Camag, Switzerland) system with nicotine (99.3% purity) as standard. Nicotine standard was run along with solid fraction and similar peaks were observed. Chromatographic separation was performed on Merck TLC plates precoated with silica gel 60 F_254_ (20 cm × 10 cm with 200 *μ*m layer thickness) from E. Merck, Germany. Standard solution (0.5 *μ*L and 3 *μ*L) was applied onto the plates as a band with 8 mm width using Camag 100 *μ*L sample syringe (Hamilton, Switzerland) using Camag Linomat 5 applicator (Camag, Switzerland). Linear ascending development was carried out in a twin trough glass chamber (20 cm × 10 cm) with toluene : ethyl acetate : formic acid : methanol (14 : 10 : 2 : 1) for nicotine standard. The chamber was previously saturated with mobile phase vapour for 25 min at room temperature (25 ± 2°C) and plates were developed at distance of approximately 80 mm from the point of application. Scanning was performed using Camag TLC scanner 3 (at 254 nm and 366 nm) through fluorescence mode and operated by win CATS software (version 1.4.1, Camag). Extracts deposited on the silica plates were visualized under ultraviolet (254 and 366 nm) and visible light.

### 2.4. Ticks

Fully engorged adult female* R. (B.) annulatus* were collected from the naturally infested calves with a history of no prior exposure to any conventional acaricides. They were washed with distilled water, dried on an absorbent paper, and used for adult immersion test (AIT).

### 2.5. Adult Immersion Test (AIT)

Adult immersion test was performed based on Drummond et al. [16]. The different concentrations of extract (10% to 1.25%) and nicotine standard (20000 ppm to 1250 ppm) were prepared in unrefined coconut oil and methanol, respectively. Four replicates, each with six ticks, were used for each concentration. The groups of six ticks selected randomly based on the size were weighed before the experiments and were immersed for two minutes in the respective dilution in a 50 mL beaker containing 10 mL extract. Ticks were recovered from the solution, dried using absorbent paper, and placed in a separate plastic specimen tube (25 mm × 50 mm). The tubes were incubated at 28 ± 2°C temperature and 80% relative humidity in a biological oxygen demand (BOD) incubator. The adult tick mortality was observed up to 15th day posttreatment. After oviposition, the eggs laid by the female ticks were collected and weighed. The eggs were kept under the same incubation conditions in a BOD incubator for the next 30 days.

The index of egg laying (IF) and per cent inhibition of fecundity (InF) were calculated (FAO 2004) as follows:

(1) Index  of  egg  laying  IF=weight  of  eggs  laid  gweight  of  female  ticks  g,Per⁡  cent  inhibition  of  fecundity  %  InF  =IF  control−IF  treated×100IF  control.

The hatching of eggs was observed visually.

### 2.6. Statistical Analysis

The analysis of data was performed [17]. Data were expressed as the standard mean ± standard error of mean (SEM). The groups were compared using one-way analysis of variance (ANOVA) for repeated measurements using statistical package for the social science (SPSS) software. Duncan's test was used for post hoc analysis in order to understand significance levels for the difference between the groups of means. A value of *P* < 0.05 was considered significant.

### 2.7. Probit Analysis

Dose response data were analysed by probit method [18] using computer programme Graph Pad-Prism-4. The median lethal concentration (LC_50_) value of petroleum ether extract of leaves of* T. leucostaphylum* against* R. (B.) annulatus* was determined by applying regression equation analysis to the probit transformed data of mortality.

## 3. Results 

**Figure 2:**
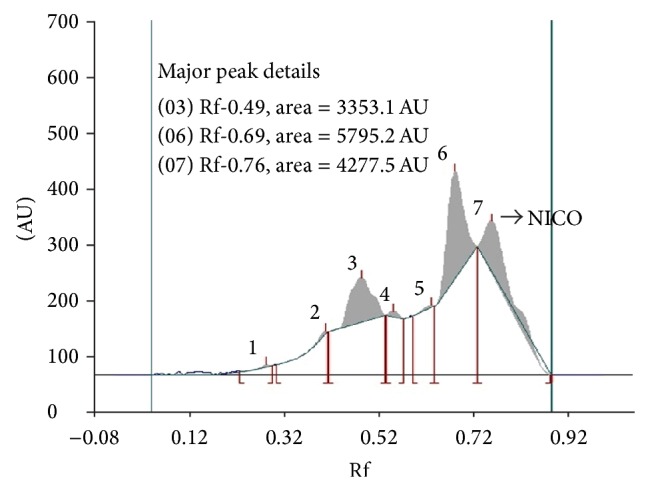
Chromatogram showing the HPTLC profiling of petroleum ether leaf extract of* T. leucostaphylum* (AU—Absorbance units; Rf—Retardation factor; NICO—Nicotine).

**Figure 3:**
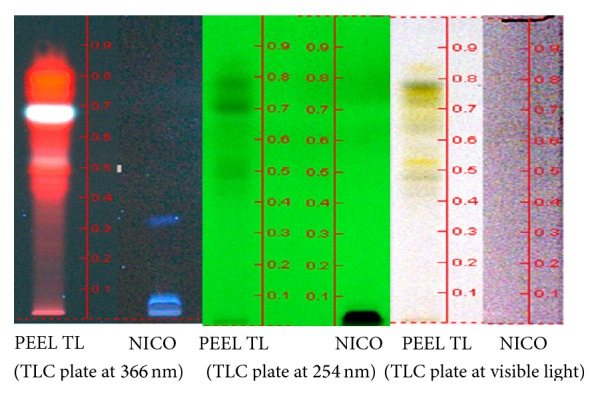
General HPTLC finger print profile of nicotine standard (NICO) with petroleum ether leaf extract of* T. leucostaphylum* (PEELTL) at wavelength 254 nm, 366 nm and visible light.

The extractive yield of petroleum ether extract of leaves of* T. leucostaphylum* leaves was 2.99%. In the present study, the HPTLC profiling revealed the presence of seven polyvalent phytoconstituents. Among the seven polyvalent components, nicotine was identified as one of the components. The Rf values ranged from 0.29 to 0.76. It is also clear from the chromatogram as shown in Figure 2 that out of 7 components the components with Rf values 0.49, 0.69, and 0.76 were found to be more predominant with an area of 3353.1, 5795.2, and 4277.5 AU, respectively. The Rf value of nicotine used as marker was 0.76 with a peak area of 1014.5 AU (Figure 3). The concentration of the alkaloid nicotine present in the extract was approximately 5.4%.

**Figure 4:**
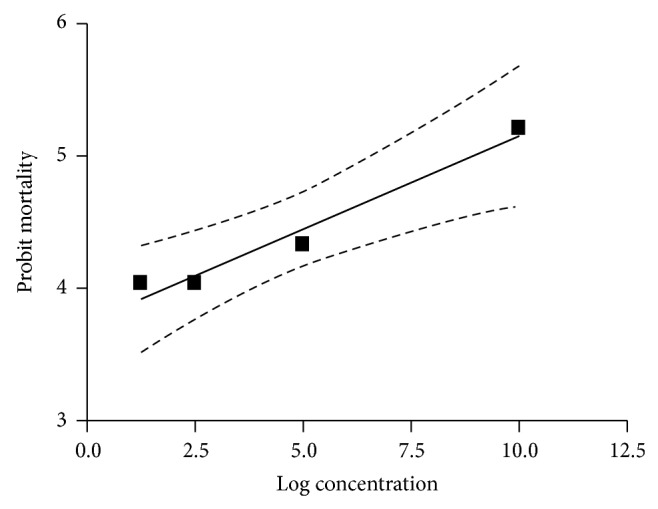
Dose mortality curve of* R. (B.) annulatus* against petroleum ether extract of leaves of* T. leucostaphylum*.

The results of acaricidal efficacy of petroleum ether extract of leaves of* T. leucostaphylum* against* R. (B.) annulatus* are summarised in Table 1. The mortality of engorged female ticks, inhibition of fecundity, and hatching of eggs were concentration dependent. The per cent adult tick mortality caused varied from 16.66 to 58.32% when tested at concentrations ranging from 1.25 to 10%. Peak mortality was observed after day 5 of treatment. No mortality was recorded in the control (unrefined coconut oil treated) group. Prior to death, ticks were swollen and black in color and they did not lay eggs. The inhibition of fecundity ranged from 12.73 to 88.97% on the treated ticks, with maximum inhibition at 10% concentration. At 10% concentration, only 50% eggs laid by treated ticks hatched. The results of probit analysis are shown in Figure 4. The LC_50_ value of the extract against* R. (B.) annulatus* was 10.46%.

In the present study, the pure standard nicotine (99.3%) was also tested for acaricidal activity at concentration ranging from 1250 to 20000 ppm. However, nicotine did not exhibit any acaricidal activity even at the higher concentration of 20000 ppm (2%) (Table 2).

## 4. Discussion

The HPTLC results indicated the presence of seven polyvalent compounds in the petroleum ether leaf extract. One or more of these polyvalent compounds could act as bioactive agent. Many researchers had previously reported the identification of bioactive compounds in the crude plant extracts [19–23]. The isolation and characterisation of these bioactive compounds could be used to formulate new herbal drugs to treat various ailments.

Further in the present study, the HPTLC profiling also detected the presence of alkaloid nicotine in the leaf extract of* T. leucostaphylum*. However, the presence of phenolic compounds (catechin and phenolic acid) and alkaloids (nicotine and caffeine) in the bark and root extracts of* T. leucostaphylum* (Dennst.) Alston. ex Mabb. is already reported [24]. Recently, the presence of high content of alkaloid, phytosteroids, saponin, and cardiac glycosides was qualitatively detected in the leaves of* T. leucostaphylum* [25]. Besides, flavonoids [26], essential oils [27], and organic acids [28] were also reported in the genus* Tetrastigma* species.

In this study, the acaricidal activities of the petroleum ether extract of* T. leucostaphylum* leaves were evaluated against* R. (B.) annulatus*. It was observed that the extract exhibited concentration-dependent effect on tick mortality and inhibition of fecundity. At the highest concentration, it also partially blocked the hatching of eggs laid by the treated ticks. As per our knowledge, there were no studies on the effects of* T. leucostaphylum* leaf extracts on adult* R. (B.) annulatus* ticks. However, the activities of other plant extracts against* R. (B.) annulatus* have been reported [11–15]. The acaricidal activity seen may be due to one or more bioactive compounds present in the leaf extract. The bioactive plant compounds such as phenolics, terpenoids, coumarins, and alkaloids were known to possess insecticidal, growth inhibiting, antimolting, and repellent activities [3]. The potential role of flavones/flavonoids in modulating the reproductive functions of ticks was already reported by Juliet et al. [13]. Alkaloid in the plant extracts causes mortality and inhibition of fecundity due to its neurotoxic properties [29]. Even though the alkaloid nicotine was quantified in the plant leaf extract,* in vitro* studies with pure compound nicotine did not have any acaricidal action against ticks at higher concentration of 2% tested. Previously, nicotine up to 1000 ppm was also tested for acaricidal activity [15]. Nicotine acts as an agonist for the nicotinic acetylcholine (nAch) receptor. Overstimulation of insect's nervous system leads to poisoning and death.Further, nicotine kills the insects rapidly within an hour causing the intensive tremors, convulsions, and then paralysis [30].

From the results, it could be speculated that nicotine has more affinity for the insect's nAch receptor than the tick receptor. However, it is indicative that a synergistic or additive action of the bioactive components may be involved in the tick mortality and inhibition of the oviposition. Based on the above observation, further detailed studies are essential for the identification of acaricidal active compound(s).

## 5. Conclusion

According to the results of the current study, it could be concluded that the petroleum ether extract of* T. leucostaphylum* leaves had acaricidal activity against adult* R. (B.) annulatus* ticks. Nicotine at higher concentration of 2% did not have any acaricidal action against ticks. Additional studies are needed to identify the active principles and their effect against ticks.

## Figures and Tables

**Table 1:** Effect of petroleum ether extract of leaves of *T*. *leucostaphylum* (PEELTL) against *R. (B.) annulatus*.

Sl. number	Acaricide	Mean tick weight per replicate ± SEM (g)	Mean % adult mortality within 15 days ± SEM	Mean eggs mass per replicate ± SEM (g)	Index of fecundity ± SEM	Inhibition of fecundity (%)	Hatching (Visual %)
1	Unrefined coconut oil	1.347 ± 0.017^ab^	0 ± 0^a^	0.575 ± 0.042^b^	0.427 ± 0.033^a^	0	100
2	PEELTL-(1.25%)	1.322 ± 0.024^a^	16.660 ± 6.80^a^	0.493 ± 0.034^b^	0.372 ± 0.024^a^	12.73	100
3	PEELTL-(2.5%)	1.342 ± 0.027^ab^	16.660 ± 9.62^a^	0.454 ± 0.044^b^	0.337 ± 0.029^a^	21.73	100
4	PEELTL-(5%)	1.341 ± 0.001^ab^	24.990 ± 8.33^a^	0.428 ± 0.043^b^	0.319 ± 0.033^a^	25.57	100
5	PEELTL-(10%)	1.320 ± 0.055^a^	58.320 ± 14.43^b^	0.059 ± 0.052^a^	0.047 ± 0.041^a^	88.97	50

*n* = 4; values are mean ± SEM; means bearing different superscripts a or b (*P* < 0.05) indicate significant difference when compared with the control and petroleum ether extract of leaves of *T*. *leucostaphylum*.

**Table 2:** Effect of nicotine against *R. (B.) annulatus*.

Sl. number	Acaricide	Mean tick weight per replicate ± SEM (g)	Mean % adult mortality within 15 days ± SEM	Mean eggs mass per replicate ± SEM (g)	Index of fecundity ± SEM	Inhibition of fecundity (%)	Hatching (Visual %)
1	Methanol (Control)	0.834 ± 0.009^a^	0 ± 0	0.390 ± 0.002^b^	0.462 ± 0.006^a^	0	100
2	1250 ppm	0.867 ± 0.011^a^	0 ± 0	0.332 ± 0.005^a^	0.383 ± 0.004^b^	16.96	100
3	2500 ppm	0.826 ± 0.021^a^	0 ± 0	0.311 ± 0.015^a^	0.377 ± 0.013^b^	18.43	100
4	5000 ppm	0.839 ± 0.016^a^	0 ± 0	0.310 ± 0.027^a^	0.369 ± 0.029^b^	20.01	100
5	10000 ppm	0.845 ± 0.016^a^	0 ± 0	0.306 ± 0.018^a^	0.364 ± 0.024^b^	21.22	100
6	20000 ppm	0.834 ± 0.006^a^	0 ± 0	0.299 ± 0.002^a^	0.358 ± 0.003^b^	22.41	100

*n* = 4; values are mean ± SEM; means bearing different superscripts a or b (*P* < 0.05) indicate significant difference when compared with the control and nicotine standard.
